# Fibrinogen-like protein 2 regulates inflammatory and metabolic reprogramming of airway smooth muscle cells through PI3K/Akt activation

**DOI:** 10.3389/fmed.2026.1802584

**Published:** 2026-05-12

**Authors:** Bo Zhao, Yanran Che

**Affiliations:** Department of Pediatrics, Shanghai Pudong New Area People's Hospital, Pudong, Shanghai, China

**Keywords:** airway smooth muscle cells, FGL2, inflammation, PDGF-BB, PI3K/Akt pathway

## Abstract

**Background:**

Fibrinogen-like protein 2 (FGL2) participates in inflammatory and immune regulation; however, its contribution to airway smooth muscle cell (ASMC) dysfunction remains poorly defined. This study investigated the role of FGL2 in platelet-derived growth factor-BB (PDGF-BB)-induced pathological responses in ASMCs and explored the underlying signaling mechanisms.

**Methods:**

Human ASMCs were transfected with FGL2-specific small interfering RNA (si-FGL2) or negative control siRNA (si-NC) and subsequently stimulated with PDGF-BB, with or without the phosphoinositide 3-kinase/protein kinase B (PI3K/Akt) pathway activator insulin-like growth factor-1 (IGF-1). Activation of the PI3K/Akt signaling, ASMC proliferation and migration, extracellular matrix (ECM) protein expression, inflammatory cytokine production, oxidative stress and antioxidant capacity, and glycolytic metabolism were systematically assessed using molecular and biochemical approaches.

**Results:**

PDGF-BB stimulation markedly increased FGL2 expression in ASMCs. Silencing FGL2 significantly attenuated PDGF-BB-induced PI3K/Akt activation, as indicated by reduced phosphorylation of PI3K and Akt. FGL2 knockdown suppressed ASMC proliferation, migration, and ECM protein expression, including collagen I, *α*-smooth muscle actin, and fibronectin. In addition, FGL2 depletion significantly reduced the expression and secretion of proinflammatory cytokines (TNF-*α*, IL-1β, and IL-6), decreased intracellular reactive oxygen species accumulation and lipid peroxidation, and restored the activities of antioxidant enzymes superoxide dismutase and glutathione peroxidase. Furthermore, glycolytic reprogramming induced by PDGF-BB-reflected by increased glucose consumption, lactate production, ATP generation, and upregulation of pyruvate kinase M2 (PKM2) and lactate dehydrogenase A (LDHA), was markedly inhibited by FGL2 knockdown. Pharmacological reactivation of PI3K/Akt signaling with IGF-1 partially reversed these inhibitory effects.

**Conclusion:**

These findings suggest that FGL2 may contribute to PDGF-BB-induced ASMC dysfunction, including enhanced proliferation, migration, inflammation, oxidative stress, and glycolytic reprogramming, potentially by modulating the PI3K/Akt signaling pathway. Although preliminary, these results indicate that FGL2 could represent a potential therapeutic target for limiting airway remodeling in chronic airway diseases and warrant further investigation.

## Introduction

Airway remodeling is a hallmark of chronic airway diseases such as asthma and chronic obstructive pulmonary disease, characterized by enhanced proliferation, migration, and extracellular matrix (ECM) deposition of airway smooth muscle cells (ASMCs), which contribute to airway narrowing and hyperresponsiveness (1, 2). Platelet-derived growth factor-BB (PDGF-BB) is recognized as a potent mitogen and chemoattractant for ASMCs, driving pathological changes associated with airway remodeling through activation of intracellular signaling cascades (3). Among these pathways, the phosphoinositide 3-kinase (PI3K)/Akt axis plays a central role in mediating cell survival, proliferation, and metabolic reprogramming in response to growth factor stimulation (4, 5).

Fibrinogen-like protein 2 (FGL2) is an immunomodulatory molecule implicated in coagulation, inflammation, and immune regulation in various pathological contexts (6). Emerging evidence suggests that FGL2 may influence cellular functions beyond hemostasis, including modulation of inflammatory responses and signal transduction pathways that overlap with those activated by PDGF-BB (7). However, the contribution of FGL2 to ASMC pathological behaviors and the underlying mechanisms remain poorly understood.

Chronic airway inflammation and oxidative stress are key drivers of airway structural changes (8). PDGF-BB stimulation induces the expression of pro-inflammatory cytokines, such as tumor necrosis factor-*α* (TNF-α), interleukin-1β (IL-1β), and interleukin-6 (IL-6), which perpetuate local inflammation and exacerbate tissue damage (9). In parallel, elevated intracellular reactive oxygen species (ROS) and disrupted antioxidant defenses contribute to oxidative damage in ASMCs, further promoting disease progression (10).

In addition to proliferative and inflammatory alterations, metabolic reprogramming, particularly enhanced glycolysis, has been increasingly recognized in proliferative cell populations, including smooth muscle cells (11). Enhanced glucose uptake, lactate production, and ATP generation support the energetic and biosynthetic demands of proliferating cells, and key glycolytic enzymes, such as pyruvate kinase M2 (PKM2) and lactate dehydrogenase A (LDHA), are upregulated in response to mitogenic stimuli (12). The PI3K/Akt pathway not only regulates growth and survival but also influences metabolic pathways, linking signaling events to metabolic adaptations (13).

Given the multifaceted roles of FGL2 in immune regulation and emerging links to signaling pathways involved in proliferation, inflammation, oxidative stress, and metabolism, we hypothesized that FGL2 may modulate PDGF-BB-induced pathological responses in ASMCs via the PI3K/Akt pathway. In this study, we investigated the effects of FGL2 knockdown on ASMC proliferation, migration, expression of inflammatory cytokines, oxidative stress, and glycolytic activity, and explored the involvement of PI3K/Akt signaling, with the aim of revealing potential therapeutic targets for airway remodeling.

## Materials and methods

### Cell culture and treatment

Human airway smooth muscle cells (ASMCs) were purchased from ScienCell Research Laboratories (Carlsbad, CA, United States). ASMCs were cultivated in high-glucose DMEM (Gibco, Waltham, MA, United States) and 10% FBS (Gibco) at 37 °C with 5% CO_2_. ASMCs were divided into four groups: (i) Control group: conventional culture; (ii) Model group (PDGF-BB + si-NC): cells were transfected with si-NC for 24 h and then treated with PDGF-BB (20 ng/mL) for a further 24 h; (iii) FGL2 silencing group (PDGF-BB + si-FGL2): cells were transfected with si-FGL2 for 24 h and then stimulated with PDGF-BB for 24 h. (iv) Pathway inhibitor group (PDGF-BB + si-FGL2 + IGF-1): cells were transfected with si-FGL2 for 24 h and then co-treated with IGF-1 (100 ng/mL) and PDGF-BB (20 ng/mL) for 24 h.

### Cell transfection

ASMCs in the logarithmic growth phase were seeded in 6-well plates (1.0 × 10^5^ cells/well) and cultured until 70–80% confluence. FGL2 siRNA (si-FGL2) or its negative control (si-NC) was introduced into ASMCs using Lipofectamine 2000 (Invitrogen, Carlsbad, CA, United States). Transfection efficiency was verified using RT-qPCR and western blotting 48 h after transfection. The siRNA sequences are listed in Table 1.

**Table 1:** siRNA sequences for human FGL2 used in this investigation.

Genes	Sense strand (5′-3′)	Antisense strand (5′-3′)
si-FGL2-1	AUUAGAUAACGAAUACCUGGA	CAGGUAUUCGUUAUCUAAUAG
si-FGL2-2	ACAUCAUUUUUUAGCUAUGAA	CAUAGCUAAAAAAUGAUGUCU
si-FGL2-3	UCUCAAUAUUGCUAAUUGCUU	GCAAUUAGCAAUAUUGAGAAU

FGL2, fibrinogen-like protein 2.

### MTT cell viability assay

Cell viability was assessed using the MTT assay. Briefly, ASMCs were seeded into 96-well plates at a density of 1 × 10^4^ cells per well and allowed to adhere under standard culture conditions. After the indicated treatments, MTT solution (1 mg/mL; M1020, Solarbio, Beijing, China) was added to each well, and the plates were incubated to allow the formation of formazan crystals. Subsequently, the supernatant was carefully removed, and the crystals were solubilized according to the manufacturer’s instructions. Absorbance was measured at 570 nm using a microplate reader. Cell viability was expressed as a percentage relative to that of the control group.

### Cell migration assay

Cell migration was evaluated using Transwell inserts (BD Biosciences, San Diego, CA, United States). Briefly, cells were resuspended in a serum-free medium, and 5 × 10^4^ cells in a total volume of 500 μL were seeded into the upper chamber of each Transwell insert. The lower chamber was filled with culture medium as a chemoattractant. After incubation for the indicated period, non-migrated cells on the upper surface of the membrane were gently removed, and the cells that had migrated to the lower surface were fixed and stained according to standard procedures. Migrated cells were visualized and counted under a light microscope at 100 × magnification. The extent of cell migration was quantified by counting cells in multiple randomly selected fields per insert.

### Enzyme-linked immunosorbent assay (ELISA)

The culture media of ASMCs were collected after the indicated treatments and clarified by centrifugation to remove cellular debris. The concentrations of tumor necrosis factor-*α* (TNF-α; DTA00D), interleukin-1β (IL-1β; DLB50), and interleukin-6 (IL-6; D6050B) were quantified using commercially available ELISA kits (R&D Systems, Minneapolis, MN, United States) according to the manufacturer’s instructions. Briefly, standards and samples were added to pre-coated microplates and incubated as specified. After washing, enzyme-linked detection antibodies and substrate solutions were applied sequentially. The reaction was stopped, and the absorbance was measured using a microplate reader. Cytokine concentrations were calculated from standard curves and expressed in pg./mL.

### Intracellular reactive oxygen species measurement

Intracellular reactive oxygen species (ROS) levels were assessed using dihydroethidium (DHE; S0063, Beyotime, Shanghai, China). Cells were incubated with the DHE working solution prepared according to the manufacturer’s instructions under dark conditions. After incubation, the cells were washed with phosphate-buffered saline to remove the excess probe. Fluorescence images were captured using a fluorescence microscope at 200 × magnification. The fluorescence intensity, which reflects intracellular ROS levels, was quantified from multiple randomly selected fields using image analysis software and normalized to the control group.

### Oxidative stress measurement

Oxidative stress in ASMCs was evaluated by measuring malondialdehyde (MDA) content and the activities of antioxidant enzymes. Briefly, ASMCs were harvested after the indicated treatments, and cell lysates were prepared according to standard procedures. MDA levels were determined using a commercial assay kit (S0131S, Beyotime, Shanghai, China) as an index of lipid peroxidation. Superoxide dismutase (SOD) activity was measured using an SOD assay kit (S0109, Beyotime), and glutathione peroxidase (GPx) activity was assessed using a GPx assay kit (S0056, Beyotime), following the manufacturer’s instructions. Absorbance values were recorded using a microplate reader, and MDA content and enzyme activities were calculated according to the provided formulas and normalized to the total protein concentration.

### Evaluation of glycolysis

Glycolysis in ASMCs was evaluated by assessing glucose consumption, lactate production, and intracellular ATP levels. After the indicated treatments, culture medium and cell lysates were collected. Glucose consumption was determined using a glucose assay kit (S0554S, Beyotime, Shanghai, China), according to the manufacturer’s instructions. Lactate secretion in the culture medium was measured using a lactic acid assay kit (S0208S, Beyotime). Intracellular ATP levels were quantified in ASMC lysates using an ATP assay kit (S0026, Beyotime). Absorbance or luminescence signals were recorded using a microplate reader, and the results were calculated based on standard curves and normalized to protein content. These parameters were collectively used to reflect the glycolytic activity of ASMCs (14).

### Reverse transcription-quantitative polymerase chain reaction

Total RNA was extracted from ASMCs after the indicated treatments using a standard RNA isolation protocol. RNA purity and concentration were assessed spectrophotometrically. Complementary DNA (cDNA) was synthesized from equal amounts of total RNA using a reverse transcription kit, according to the manufacturer’s instructions. Quantitative PCR was subsequently performed using a real-time PCR system with gene-specific primers. The primer sequences used in this study are listed in Table 2. Amplification conditions were set according to standard protocols, and each sample was analyzed in triplicate. Relative mRNA expression levels were calculated using the 2^−ΔΔCt^ method after normalization to an internal reference gene.

**Table 2:** Primer oligonucleotide sequences used in this study.

Genes	Forward primer (5′-3′)	Reverse primer (5′-3′)
Human FGL2	CCAAGCACTTTAAGCCATAAATC	GGAATTAATTGCCCTATTAGATAACG
Human TNF-α	CTGGGCAGGTCTACTTTGGG	CTGGAGGCCCCAGTTTGAAT
Human IL-1β	GCTCGCCAGTGAAATGATGG	TCGTGCACATAAGCCTCGTT
Human IL-6	GTAGTGAGGAACAAGCCAGAG	TACATTTGCCGAAGAGCC
Human Collagen I	GAGGGCCAAGACGAAGACATC	CAGATCACGTCATCGCACAAC
Human α-SMA	AAAGCAAGTCCTCCAGCGTT	TTAGTCCCGGGGATAGGCAA
Human Fibronectin	GATACCATCATCCCAGCTGTTC	CAGGAAGTTGGTTAAATCAATGGA
Human GAPDH	CATGTTGCAACCGGGAAGGA	CGCCCAATACGACCAAATCAG

FGL2, fibrinogen-like protein 2; TNF-α, tumor necrosis factor-alpha; IL-1β, interleukin-1 beta; IL-6, interleukin-6; α-SMA, alpha-smooth muscle actin; GAPDH, glyceraldehyde 3-phosphate dehydrogenase.

### Western blotting

Protein expression in ASMCs was analyzed using western blotting. Briefly, ASMCs were harvested after the indicated treatments and lysed in ice-cold lysis buffer containing protease and phosphatase inhibitors. Protein concentrations were determined using a standard protein assay. Equal amounts of protein were separated by SDS-polyacrylamide gel electrophoresis and transferred onto polyvinylidene difluoride (PVDF) membranes. The membranes were blocked with 5% non-fat milk or bovine serum albumin and then incubated overnight at 4 °C with the following primary antibodies: FGL2 (1:500, sc-100276, mouse monoclonal, Santa Cruz Biotechnology); phosphorylated PI3K (p85, Tyr458; 1:400, ab278545, rabbit monoclonal, Abcam); total PI3K (1:1000, ab302958, rabbit monoclonal, Abcam); phosphorylated Akt (Ser473; 1:1000, ab81283, rabbit monoclonal, Abcam); total Akt (1:800, ab8805, rabbit polyclonal, Abcam); PKM2 (1:1000, K000067M, mouse monoclonal, Solarbio); LDHA (1:1000, sc-137243, mouse monoclonal, Santa Cruz Biotechnology); and GAPDH (1:2000, ab8245, mouse monoclonal, Abcam). After washing, membranes were incubated with the appropriate horseradish peroxidase-conjugated secondary antibodies. Protein bands were visualized using an enhanced chemiluminescence detection system and quantified by densitometric analysis. Target protein expression levels were normalized to GAPDH, and phosphorylated protein levels were normalized to their corresponding total proteins.

### Statistical analysis

Data are expressed as mean ± standard deviation (SD). Statistical analyses were performed using the SPSS software (version 20.0; SPSS, Chicago, IL, United States). For comparisons involving a single independent variable among multiple groups, one-way analysis of variance (ANOVA) was applied, followed by *post hoc* comparisons using the least significant difference (LSD) test. For experiments involving multiple independent factors, data were analyzed using two-way analysis of variance to evaluate the main effects of each factor and their interaction effects. Where appropriate, *post hoc* multiple comparisons were performed using the LSD test. All statistical tests were two-tailed, and differences were considered statistically significant at *p* < 0.05.

## Results

### FGL2 expression is upregulated in PDGF-BB-stimulated ASMCs

**Figure 1:**
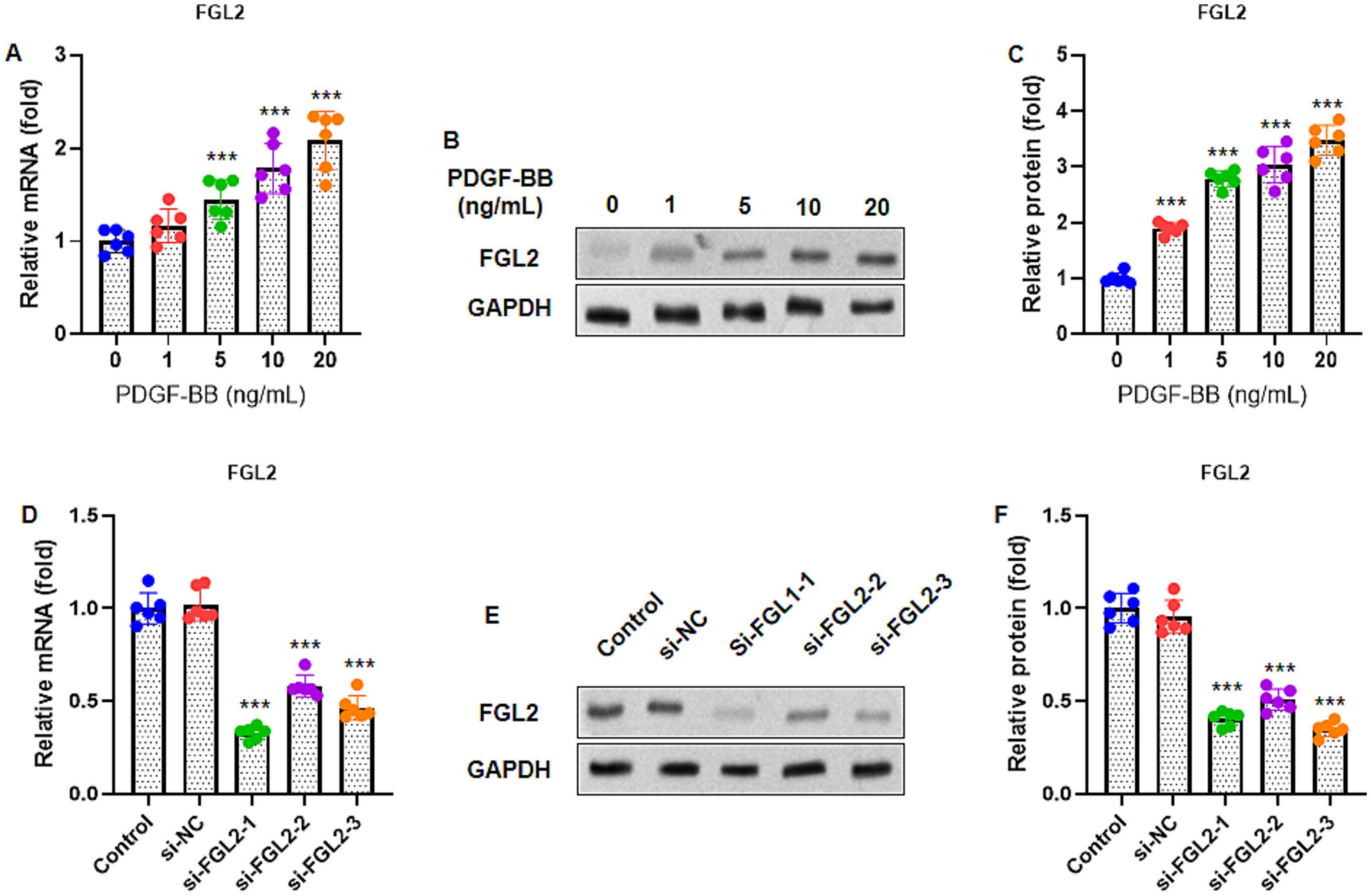
FGL2 expression was increased in airway smooth muscle cells (ASMCs) treated with PDGF-BB. **(A)** Human ASMCs were incubated with different concentrations of PDGF-BB (0, 1, 5, 10, and 20 ng/mL) for 24 h. The FGL2 mRNA expression was determined by RT-qPCR. **(B)** Representative blots of FGL2 protein by western blot. **(C)** The FGL2 protein relative expression was quantified by normalization to GAPDH. **(D)** FGL2 was knocked down in ASMCs by transfection with three sequences of FGL2 siRNA (si-FGL2) and control siRNA (si-NC). RT-qPCR was performed to detect mRNA expression of FGL2 48 h after transfection. **(E)** Representative blots of FGL2 protein by western blot. **(F)** Quantification of FGL2 protein expression by normalization to GAPDH. The #1 sequence shows the best inhibitory effect and was chosen for further experiments. Data are presented as mean ± SD in 6 duplicate wells and analyzed using ANOVA, followed by Dunnett *post hoc* analysis. ** *p* < 0.01, *** *p* < 0.001 vs. control group.

To determine whether FGL2 is involved in PDGF-BB-induced responses in ASMCs, FGL2 expression was examined at both the mRNA and protein levels. As shown in Figure 1A, treatment of human ASMCs with increasing concentrations of PDGF-BB (0, 1, 5, 10, and 20 ng/mL) for 24 h resulted in a significant, dose-dependent increase in FGL2 mRNA expression, as assessed by RT-qPCR. Western blot analysis consistently demonstrated a corresponding elevation in FGL2 protein levels following PDGF-BB stimulation (Figure 1B), and densitometric quantification confirmed a significant upregulation of FGL2 protein normalized to GAPDH (Figure 1C).

To further investigate the functional role of FGL2, ASMCs were transfected with three different FGL2-specific siRNAs (si-FGL2) or a negative control siRNA (si-NC). RT-qPCR analysis performed 48 h after transfection showed that all three siRNA sequences effectively reduced FGL2 mRNA expression compared to the control, with si-FGL2 #1 exhibiting the most pronounced knockdown efficiency (Figure 1D). This result was further validated at the protein level by western blotting (Figure 1E), and quantitative analysis confirmed a marked reduction in FGL2 protein expression following si-FGL2 #1 transfection (Figure 1F). Therefore, si-FGL2 #1 was selected for subsequent experiments.

### FGL2 knockdown suppresses PI3K/Akt signaling, proliferation, and migration in PDGF-BB-stimulated ASMCs

**Figure 2:**
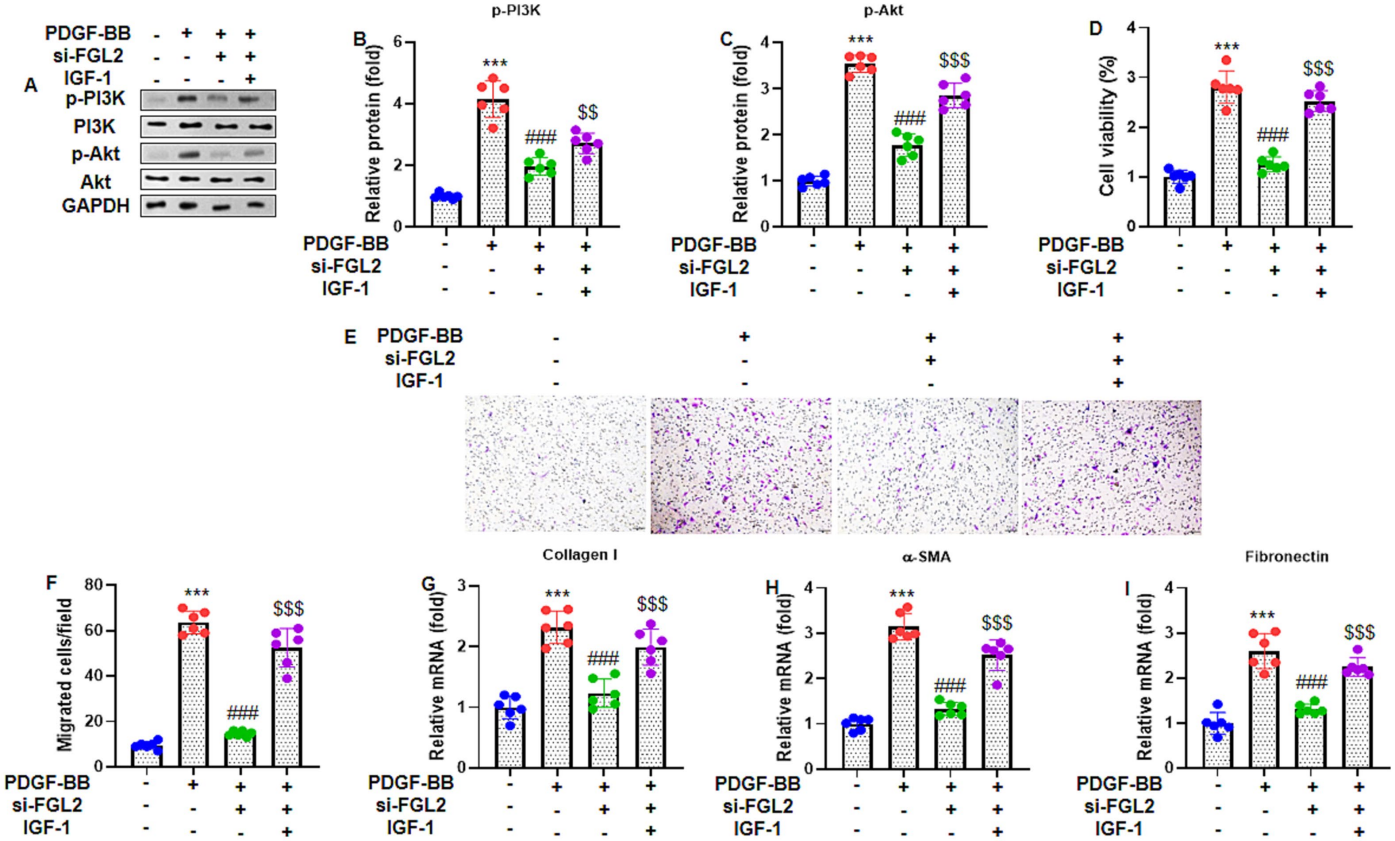
FGL2 knockdown inhibits PI3K/Akt pathway and proliferation and migration in PDGF-BB-treated ASMCs. ASMCs were transfected with si-FGL2 for 24 h, and cotreated with a PI3K/Akt pathway activator IGF-1 (100 ng/mL) and PDGF-BB (20 ng/mL) for further 24 h. **(A)** Representative gel blots of p-PI3K (p85, Tyr458, normalized to total PI3K) and p-Akt (Ser473, normalized to total Akt). **(B,C)** The relative expression of p-PI3K and p-Akt protein were quantified. **(D)** Cell viability was determined by MTT assay. **(E)** The migration ability of ASMCs were assessed by Transwell, the migrated cells were stained with 0.1% crystal violet, and the representative images are shown (magnification 100×). **(F)** Quantification of the number of migrated cells. RT-qPCR was used to assess mRNA expression of **(G)** Collagen I, **(H)** *α*-SMA and **(I)** Fibronectin. Data are presented as mean ± SD in 6 duplicate wells, analyzed by two-way ANOVA, followed by Tukey post hoc analysis for multiple comparisons. *** *p* < 0.001 vs. control group; ### *p* < 0.001 vs. PDGF-BB group; $$$ *p* < 0.001 vs. PDGF-BB + si-FGL2 group.

PDGF-BB markedly induced a fibrotic phenotype in ASMCs, as demonstrated by the significantly increased expression of ECM-related proteins, including collagen I, *α*-SMA, and fibronectin (Supplementary Figures 1A–D). In parallel, PDGF-BB robustly activated the PI3K/Akt signaling pathway, as evidenced by increased phosphorylation of PI3K (p85, Tyr458) and Akt (Ser473) (Figure 2). Silencing of FGL2 significantly attenuated PDGF-BB-induced fibrotic responses, suppressing ECM protein expression, ASMC proliferation and migration, and was accompanied by reduced PI3K and Akt phosphorylation.

Notably, pharmacological reactivation of PI3K/Akt signaling with IGF-1 partially reversed the inhibitory effects of FGL2 knockdown on ECM expression, ASMC proliferation and migration, inflammatory cytokine production, oxidative stress, and glycolytic activity, although these effects did not reach the levels observed with PDGF-BB alone. Collectively, these findings indicate that PI3K/Akt signaling functionally contributes to FGL2-mediated pathological responses in PDGF-BB-stimulated ASMCs. Given the pleiotropic nature of IGF-1, the partial rescue observed supports PI3K/Akt involvement but does not imply exclusive pathway specificity.

### FGL2 knockdown attenuates PDGF-BB-induced inflammatory responses in ASMCs

**Figure 3:**
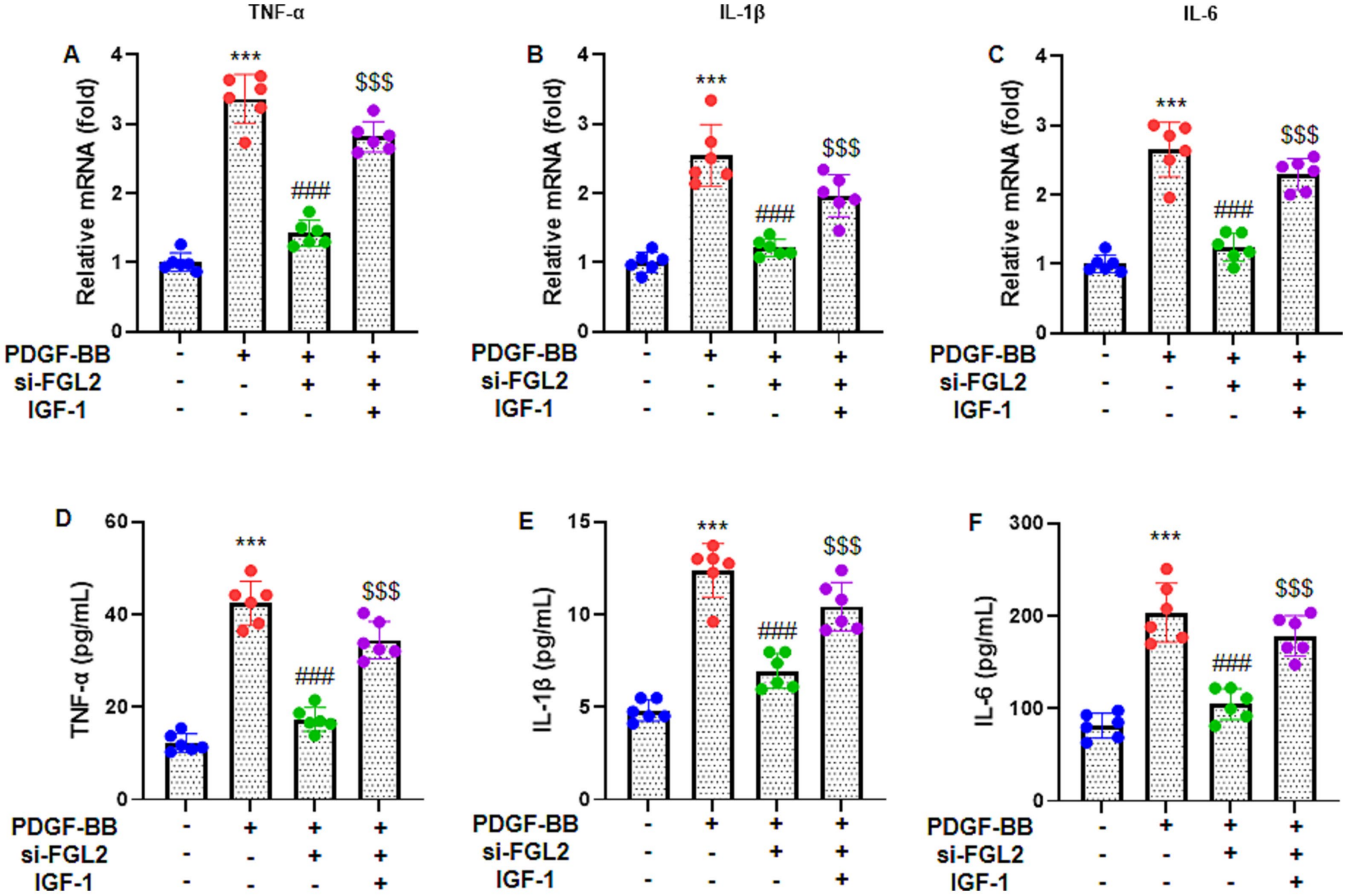
FGL2 knockdown inhibits PDGF-BB-stimulated inflammatory response. RT-qPCR was applied to determine the mRNA expression of pro-inflammatory cytokines **(A)** TNF-α, **(B)** IL-1β, and **(C)** IL-6 in ASMCs. ELISA was applied to determine the levels of **(D)** TNF-α, **(E)** IL-1β, and **(F)** IL-6 in ASMCs supernatants. Data are presented as mean ± SD in 6 duplicate wells, analyzed by two-way ANOVA, followed by Tukey post hoc analysis for multiple comparisons. *** *p* < 0.001 vs. control group; ### *p* < 0.001 vs. PDGF-BB group; $$$p < 0.001 vs. PDGF-BB + si-FGL2 group.

To determine whether FGL2 regulates PDGF-BB-induced inflammatory responses in ASMCs, cells were transfected with si-FGL2 before PDGF-BB stimulation. RT-qPCR analysis showed that PDGF-BB significantly upregulated the mRNA expression of TNF-*α*, IL-1β, and IL-6 compared with the control group (Figures 3A–C). Consistent with this, ELISA demonstrated marked increases in the secretion of these cytokines in the culture supernatants after PDGF-BB treatment (Figures 3D–F). In parallel, western blot analysis confirmed that PDGF-BB significantly elevated TNF-*α*, IL-1β, and IL-6 protein levels (all *p* < 0.001) (Supplementary Figures 2A–D). Notably, FGL2 knockdown markedly attenuated PDGF-BB-induced increases in cytokine expression at both the transcriptional and protein levels, as well as in their secretion. Moreover, IGF-1 partially reversed the suppressive effects of si-FGL2, restoring TNF-α, IL-1β, and IL-6 expression, although levels remained lower than those in PDGF-BB-treated cells. Collectively, these findings indicate that FGL2 is a critical mediator of PDGF-BB–induced inflammatory responses in ASMCs and that IGF-1 can modulate this FGL2-dependent signaling axis.

### FGL2 knockdown reduces PDGF-BB-induced intracellular ROS and oxidative stress in ASMCs

**Figure 4:**
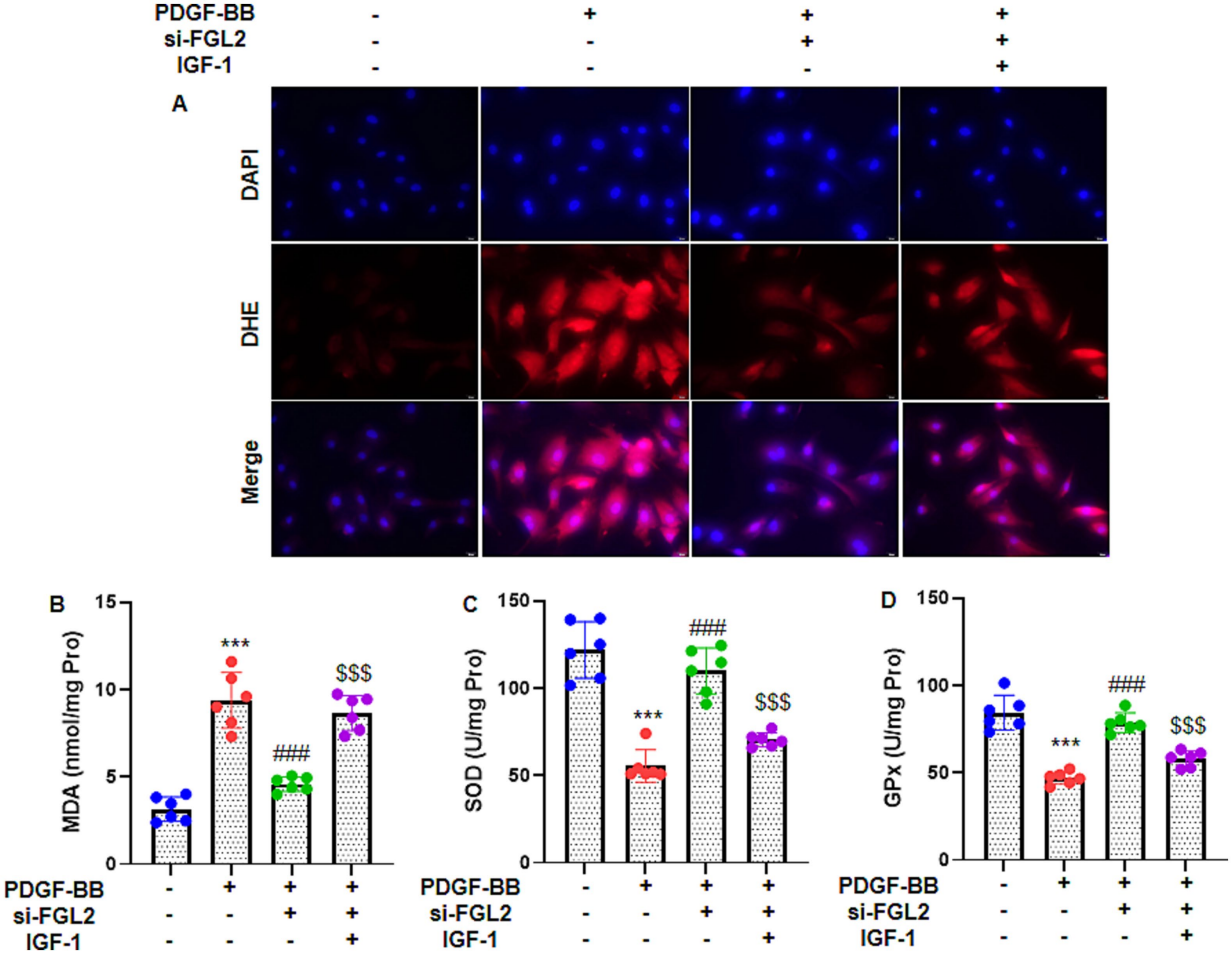
FGL2 knockdown inhibits PDGF-BB-stimulated intracellular ROS and oxidative stress in ASMCs. **(A)** Immunofluorescence and DHE staining was applied to detect intracellular ROS of ASMCs (400×). The representative image are shown. Colorimetric method was applied to measure the oxidative stress indicators: **(B)** MDA content, **(C)** SOD activity, and **(D)** GPx activity in cell lysate. Data are presented as mean ± SD in 6 duplicate wells, analyzed by two-way ANOVA, followed by Tukey post hoc analysis for multiple comparisons. *** *p* < 0.001 vs. control group; ### *p* < 0.001 vs. PDGF-BB group; $$$ *p* < 0.001 vs. PDGF-BB + si-FGL2 group.

To assess the role of FGL2 in oxidative stress, ASMCs were transfected with si-FGL2 before PDGF-BB stimulation. Immunofluorescence analysis using DHE staining revealed that PDGF-BB markedly increased intracellular ROS levels compared to control cells, whereas FGL2 knockdown significantly attenuated ROS accumulation (Figure 4A). Consistent with the ROS findings, biochemical assays of cell lysates demonstrated that PDGF-BB treatment elevated MDA content (Figure 4B) and reduced the activities of the antioxidant enzymes SOD (Figure 4C) and GPx (Figure 4D). Silencing FGL2 effectively reversed these changes, indicating reduced lipid peroxidation and enhanced antioxidant defense. Collectively, these results suggest that FGL2 contributes to PDGF-BB–induced oxidative stress in ASMCs.

### FGL2 knockdown suppresses PDGF-BB-induced glycolysis in ASMCs

**Figure 5:**
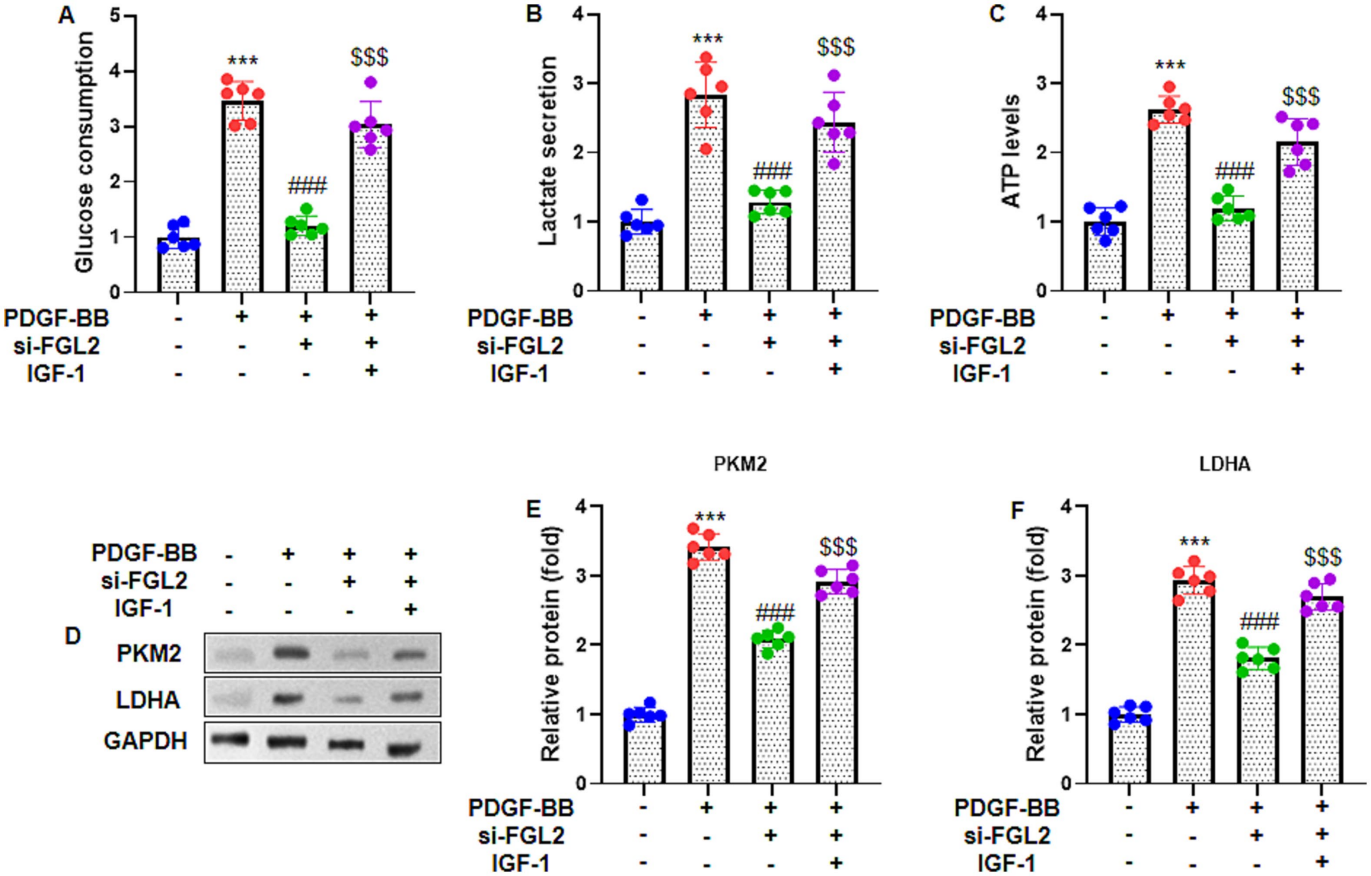
FGL2 knockdown inhibits PDGF-BB-stimulated glycolysis in ASMCs. The glycolysis-related indicators were measured, including **(A)** glucose consumption, **(B)** lactate secretion, and **(C)** ATP levels in lysate of ASMCs. **(D)** Representative bands of glycolysis-related proteins by western blot. **(E,F)** Quantification of PKM2 and LDHA in ASMCs, which were normalized to GAPDH. Data are presented as mean ± SD in 6 duplicate wells, analyzed by two-way ANOVA, followed by Tukey post hoc analysis for multiple comparisons. *** *p* < 0.001 vs. control group; ### *p* < 0.001 vs. PDGF-BB group; $$$ *p* < 0.001 vs. PDGF-BB + si-FGL2 group.

To explore the effect of FGL2 on glycolytic activity, ASMCs were transfected with si-FGL2 and stimulated with PDGF-BB. Biochemical analysis revealed that PDGF-BB significantly increased glucose consumption (Figure 5A), lactate secretion (Figure 5B), and intracellular ATP levels (Figure 5C) compared to control cells. Silencing FGL2 markedly attenuated these PDGF-BB-induced enhancements, indicating reduced glycolytic activity. At the protein level, western blot analysis demonstrated that PDGF-BB upregulated the expression of key glycolytic enzymes, PKM2 and LDHA (Figure 5D). Quantitative analysis confirmed that FGL2 knockdown significantly decreased PKM2 and LDHA protein levels normalized to GAPDH (Figures 5E,F). These results indicate that FGL2 promotes PDGF-BB-induced glycolysis in ASMCs by upregulating glycolytic enzymes.

## Discussion

In this study, we demonstrated that FGL2 knockdown significantly attenuated PDGF-BB-induced pathological responses in ASMCs, including activation of the PI3K/Akt signaling pathway, enhanced proliferation and migration, increased expression of inflammatory cytokines, oxidative stress, and glycolytic reprogramming. Collectively, these findings identify FGL2 as a critical regulator of PDGF-BB-mediated ASMC dysfunction and suggest its potential relevance as a therapeutic target for airway remodeling associated with chronic airway diseases.

PDGF-BB is widely used to model airway remodeling *in vitro* because of its potent ability to induce ASMC proliferation and migration, which are central features of asthma pathophysiology. Previous studies have established that PDGF-BB activates multiple intracellular signaling pathways, particularly PI3K/Akt, to drive these phenotypic changes in ASMCs and related smooth muscle cell types (1, 15). In agreement with these reports, we observed that PDGF-BB markedly increased PI3K and Akt phosphorylation. Importantly, FGL2 knockdown significantly reduced this activation, indicating that FGL2 positively regulates PI3K/Akt signaling under PDGF-BB stimulation.

Rather than being selected solely based on prior literature, the PI3K/Akt pathway emerged as a central mechanistic axis supported by multiple experimental observations in this study. Specifically, FGL2 silencing consistently suppressed PI3K/Akt activation in parallel with reduced ASMC proliferation, migration, inflammatory cytokine production, oxidative stress, and glycolytic activity. Furthermore, pharmacological activation of PI3K/Akt signaling using IGF-1 partially reversed these effects, providing functional evidence for pathway involvement and supporting a mechanistic link between FGL2 and PI3K/Akt signaling in PDGF-BB-induced ASMC dysfunction.

Although our data demonstrate that FGL2 knockdown significantly suppresses PDGF-BB-induced phosphorylation of PI3K and Akt, the precise molecular mechanism by which FGL2 regulates PI3K/Akt activation remains to be fully elucidated. FGL2 is a multifunctional immunoregulatory protein that has been reported to modulate cellular signaling indirectly through interactions with immune and cell-surface receptors and by shaping inflammatory microenvironments (6, 16). In this context, FGL2 may influence PI3K/Akt activation upstream of receptor tyrosine kinase signaling or through the modulation of autocrine/paracrine mediators induced by PDGF-BB stimulation. However, direct receptor binding or a canonical linear signaling cascade linking FGL2 to PI3K/Akt has not yet been established in ASMCs. Therefore, our findings support a functional upstream regulatory role of FGL2 in PI3K/Akt signaling rather than a defined direct activation mechanism. Further studies are required to identify the specific receptor(s), adaptor proteins, or intermediate signaling molecules mediating FGL2-dependent PI3K/Akt activation in ASMCs.

The PI3K/Akt pathway is a well-established regulator of cell survival, proliferation, migration, and metabolism (17). Consistent with its role in airway remodeling, inhibition of PI3K/Akt signaling has been shown to suppress PDGF-BB-induced ASMC proliferation and migration (15). In the present study, IGF-1-mediated activation of this pathway partially rescued the inhibitory effects of FGL2 knockdown on ASMC proliferation and migration, further supporting the functional contribution of PI3K/Akt signaling in FGL2-driven cellular responses.

Importantly, IGF-1 was employed as a functional pathway activator rather than a highly specific molecular tool. As a pleiotropic growth factor, IGF-1 activates multiple downstream signaling cascades, including the MAPK/ERK and mTOR pathways, in addition to PI3K/Akt signaling (18–20). Therefore, the partial phenotypic rescue observed following IGF-1 treatment should be interpreted as supportive, but not definitive, evidence of PI3K/Akt involvement. More selective genetic approaches, such as ectopic expression of constitutively active Akt (e.g., myristoylated hemagglutinin-tagged Akt1 plasmid, pcDNA3 Myr-HA-Akt1), are commonly used to achieve pathway-specific activation independent of upstream receptor signaling (21). These strategies would provide greater mechanistic specificity and will be pursued in future studies to further validate Akt-dependent signaling downstream of FGL2.

In addition to structural remodeling, chronic airway diseases are characterized by persistent inflammation, which contributes to disease progression (22). In this study, PDGF-BB increased the expression and secretion of pro-inflammatory cytokines, including TNF-*α*, IL-1β, and IL-6, in ASMCs. FGL2 knockdown significantly attenuated these responses, suggesting that FGL2 amplifies PDGF-BB-induced inflammatory signaling in ASMCs. This is consistent with previous findings implicating regulatory molecules in modulating cytokine production in airway structural cells (1, 23).

Oxidative stress is another key contributor to airway remodeling, as excessive ROS promote inflammation and smooth muscle dysfunction (24). Here, PDGF-BB increased ROS production and impaired antioxidant defenses, whereas FGL2 silencing reduced ROS accumulation and restored antioxidant enzyme activity, including SOD and GPx. These findings suggest that FGL2 may enhance oxidative stress susceptibility in ASMCs, thereby contributing to remodeling processes. This is consistent with the evidence that antioxidant pathways can modulate smooth muscle proliferation and inflammatory signaling (25).

Metabolic reprogramming, particularly enhanced glycolysis, is increasingly recognized as a hallmark of proliferative and migratory cell phenotypes (26). In the present study, PDGF-BB increased glucose uptake, lactate production, and ATP generation, accompanied by the upregulation of glycolytic enzymes, PKM2 and LDHA. These changes were significantly reversed by FGL2 knockdown, indicating that FGL2 contributes to PDGF-BB-induced glycolytic reprogramming in ASMCs. This is consistent with evidence that growth factor–driven metabolic shifts support airway remodeling, and that glycolysis inhibition can suppress pathological cellular activation (8).

Importantly, PI3K/Akt signaling is a known regulator of glycolysis and metabolic enzyme expression, providing a plausible mechanistic link between FGL2 activation, PI3K/Akt signaling, and metabolic reprogramming in ASMCs (17, 27). However, while FGL2 silencing simultaneously reduced glycolysis, oxidative stress, inflammation, and proliferative responses, this study did not directly dissect the causal relationships among these processes. Targeted metabolic, antioxidant, or anti-inflammatory rescue experiments were not performed to determine the interdependence among these pathways. Therefore, these changes should be interpreted as coordinated downstream effects of FGL2-mediated PI3K/Akt regulation rather than independent mechanistic cascades.

Collectively, our findings suggest that FGL2 functions as an upstream regulator of PDGF-BB-induced ASMC dysfunction by modulating PI3K/Akt-dependent processes, including proliferation, migration, inflammation, oxidative stress, and glycolytic reprogramming. However, it is important to emphasize that these conclusions are based entirely on *in vitro* experimental evidence using cultured human ASMCs. Therefore, while FGL2 emerges as a potentially important molecular regulator in airway smooth muscle cell biology, its translational relevance and therapeutic applicability in airway remodeling remain to be validated *in vivo*. This cautious interpretation is consistent with previous reports highlighting that in vitro ASMC models, although informative, do not fully recapitulate the complexity of airway remodeling *in vivo*, including immune epithelial stromal interactions and whole-tissue microenvironmental effects (28, 29).

### Study limitations and future directions

Despite the mechanistic insights provided by this study, several limitations should be acknowledged. First, the present work relied exclusively on an in vitro PDGF-BB-stimulated ASMC model, which does not fully recapitulate the complexity of the airway microenvironment in vivo, and no animal models, pharmacological inhibition approaches, or patient-derived airway tissues were included. Therefore, the therapeutic relevance of targeting FGL2 remains hypothetical at this stage. Future studies should incorporate airway remodeling or asthma animal models, as well as validation in clinical airway tissue samples, to establish in vivo relevance and translational significance. Second, although IGF-1 was used to functionally reactivate PI3K/Akt signaling and supported its involvement in FGL2-mediated ASMC dysfunction, its pleiotropic nature limits definitive conclusions regarding pathway specificity. More selective genetic approaches, such as constitutively active Akt constructs or pathway-specific rescue strategies, will be required to confirm Akt-dependent mechanisms. Third, although FGL2 knockdown was associated with reduced ASMC proliferation, migration, inflammation, oxidative stress, and glycolytic activity, the causal relationships among these processes were not directly dissected because of the absence of pathway-specific interventions, such as glycolytic inhibitors, reactive oxygen species scavengers, or cytokine-neutralizing strategies. Finally, unbiased transcriptomic or proteomic profiling was not performed, limiting the comprehensive identification of downstream signaling networks and leaving open the possibility that additional pathways beyond PI3K/Akt contribute to FGL2-regulated ASMC responses. Future integrative omics and targeted functional studies will be essential to delineate the broader signaling landscape and further evaluate the therapeutic potential of FGL2 in airway remodeling.

## Conclusion

**Figure 6:**
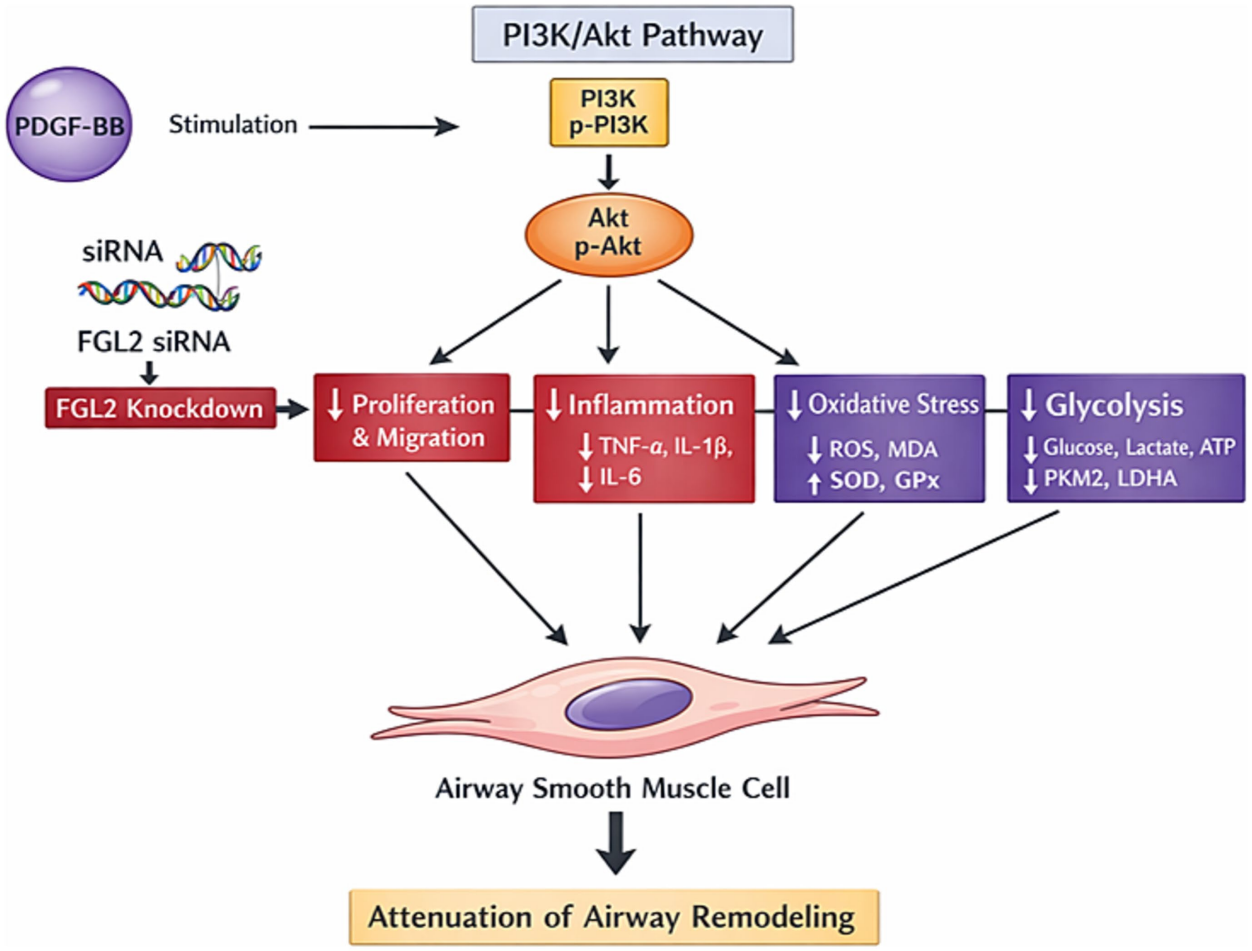
Schematic representation of FGL2-mediated regulation of PDGF-BB-induced responses in airway smooth muscle cells (ASMCs). PDGF-BB stimulation upregulates FGL2, which activates the PI3K/Akt signaling pathway. Activated PI3K/Akt promotes multiple downstream pathological processes, including proliferation and migration, inflammatory cytokine production (TNF-α, IL-1β, IL-6), oxidative stress (↑ROS, ↑MDA, ↓SOD, ↓GPx), and enhanced glycolysis (↑glucose consumption, ↑lactate production, ↑ATP generation, ↑PKM2, ↑LDHA). Knockdown of FGL2 using siRNA inhibits PI3K/Akt activation, leading to reduced ASMC proliferation, migration, inflammation, oxidative stress, and glycolytic activity, collectively contributing to the attenuation of airway remodeling. Arrows indicate stimulatory effects; downward arrows indicate reductions following FGL2 knockdown.

In conclusion, this study demonstrates that FGL2 contributes to PDGF-BB-induced ASMC dysfunction by regulating the PI3K/Akt signaling pathway and associated cellular processes, such as proliferation, migration, inflammatory responses, oxidative stress, and glycolytic metabolism (Figure 6). These findings identify FGL2 as a novel upstream modulator of airway smooth muscle cell pathology under *in vitro* conditions. However, given the lack of *in vivo* validation and clinical data, the potential of FGL2 as a therapeutic target for airway remodeling remains speculative and requires further investigation in animal models and patient-derived tissues before it can be considered for translation to the clinic.

## Data Availability

The datasets presented in this study can be found in online repositories. The names of the repository/repositories and accession number(s) can be found in the article/Supplementary material.
