# Supplementary material for: Fibrinogen-like protein 2 regulates inflammatory and metabolic reprogramming of airway smooth muscle cells through PI3K/Akt activation
Source: Front Med (Lausanne). 2026 May 12;13:1802584. doi: 10.3389/fmed.2026.1802584 (PMC13220777; doi:10.3389/fmed.2026.1802584)
Supplement: Supplementary file 1 [file Data_Sheet_1.doc]

**Supplementary Materials**


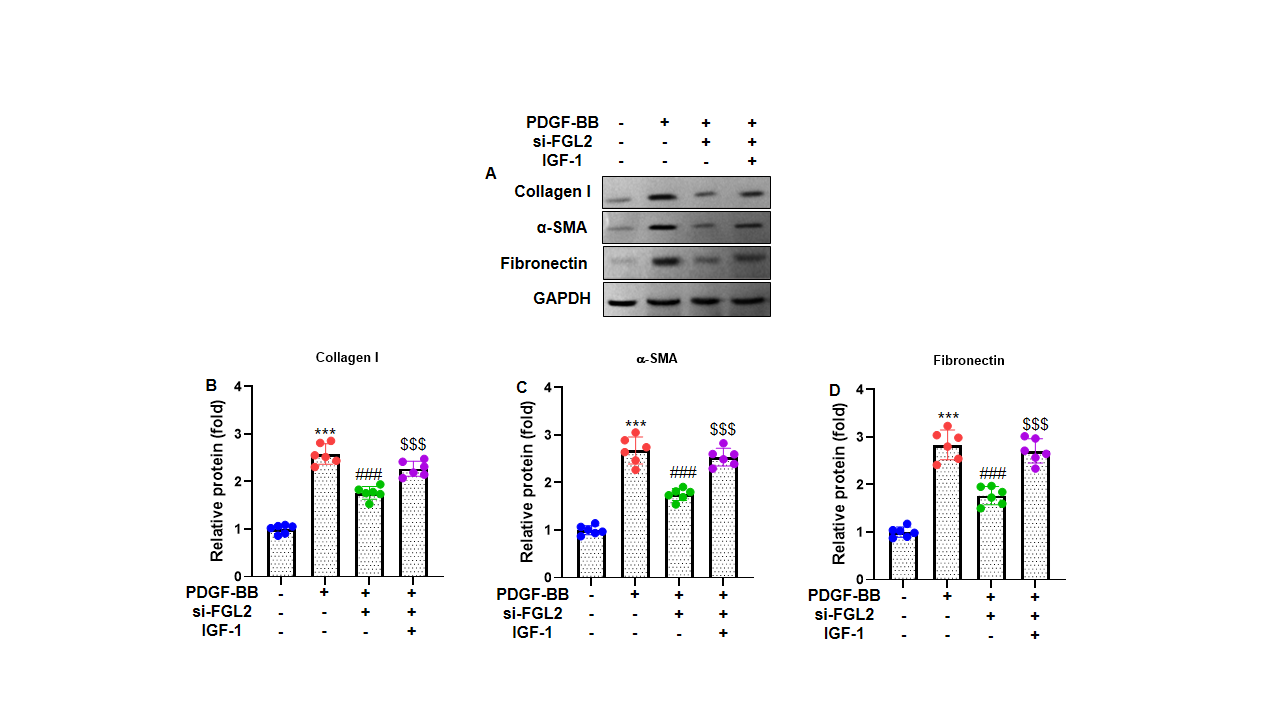


**Supplementary Figure 1.** FGL2 knockdown suppresses PDGF-BB-induced fibrotic protein expression, which is partially reversed by IGF-1. (A) Representative western blot images showing the protein expression levels of collagen I, α-SMA, and fibronectin in cells treated with PDGF-BB, si-FGL2, and/or IGF-1, with GAPDH used as a loading control. (B-D) Quantitative analysis of relative protein expression levels of (B) Collagen I, (C) α-SMA, and (D) Fibronectin. Data are presented as the mean ± SD from independent experiments. ***P < 0.001 vs. control group; ###P < 0.001 vs. PDGF-BB group; $$$P < 0.001 vs. PDGF-BB + si-FGL2 group.

**
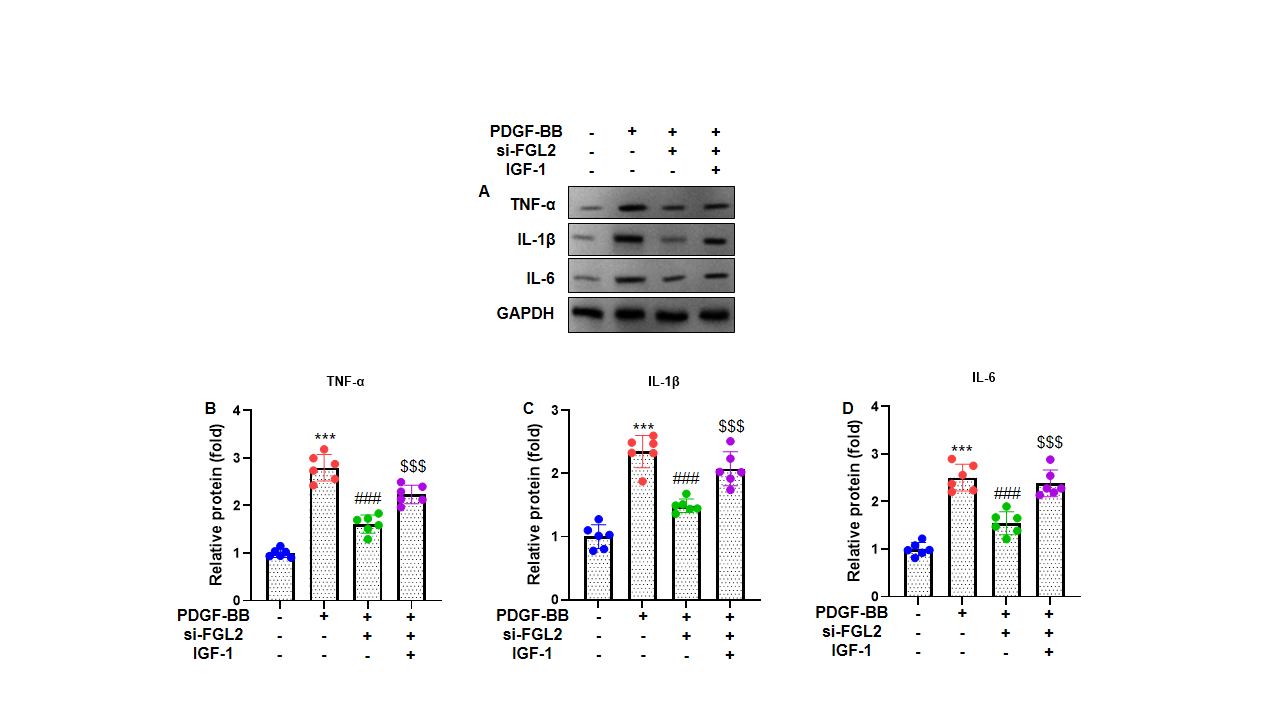
**

**Supplementary Figure 2.** Effects of PDGF-BB, FGL2 knockdown, and IGF-1 on the expression of proinflammatory cytokines. (A) Representative western blot images showing the protein expression levels of TNF-α, IL-1β, and IL-6 under control conditions, PDGF-BB stimulation, PDGF-BB with si-FGL2, and PDGF-BB with si-FGL2 plus IGF-1 treatment. GAPDH was used as a loading control. (B-D) Quantitative analysis of TNF-α (B), IL-1β (C), and IL-6 (D) protein expression normalized to GAPDH and expressed as fold change relative to the control group. Data are presented as mean ± SD (n = 6). ***P < 0.001 vs. control group; ###P < 0.001 vs. PDGF-BB group; $$$P < 0.001 vs. PDGF-BB + si-FGL2 group.
